# Clinical utility of cerebrospinal fluid biomarkers measured by LUMIPULSE^®^ system

**DOI:** 10.1002/acn3.51681

**Published:** 2022-11-02

**Authors:** Hisashi Nojima, Satoshi Ito, Akira Kushida, Aki Abe, Wataru Motsuchi, David Verbel, Manu Vandijck, Geert Jannes, Ina Vandenbroucke, Katsumi Aoyagi

**Affiliations:** ^1^ FUJIREBIO Inc. 2‐1‐1, Nishishinjuku, Shinjuku‐ku Tokyo 163‐0410 Japan; ^2^ Eisai Co., Ltd. 4‐6‐10 Koishikawa Bunkyo‐ku Tokyo 112‐8088 Japan; ^3^ Eisai Inc. 200 Metro Boulevard Nutley New Jersey 07110 USA; ^4^ Fujirebio‐Europe N.V. Technologiepark 6 9052 Ghent Belgium

## Abstract

**Objectives:**

Cerebrospinal fluid (CSF) biomarkers of Alzheimer's disease (AD) are well‐established in research settings, but their use in routine clinical practice remains a largely unexploited potential. Here, we examined the relationship between CSF biomarkers, measured by a fully automated immunoassay platform, and brain β‐amyloid (Aβ) deposition status confirmed by amyloid positron emission tomography (PET).

**Methods:**

One hundred ninety‐nine CSF samples from clinically diagnosed AD patients enrolled in a clinical study and who underwent amyloid PET were used for the measurement of CSF biomarkers Aβ 1–40 (Aβ40), Aβ 1–42 (Aβ42), total tau (t‐Tau), and phosphorylated tau‐181 (p‐Tau181) using the LUMIPULSE system. These biomarkers and their combinations were compared to amyloid PET classification (negative or positive) using visual read assessments. Several combinations were also analyzed with a multivariable logistic regression model.

**Results:**

Aβ42, t‐Tau, and p‐Tau181, and the ratios of Aβ42 with other biomarkers had a good diagnostic agreement with amyloid PET imaging. The multivariable logistic regression analysis showed that amyloid PET status was associated with Aβ40 and Aβ42, but other factors, such as MMSE, sex, t‐Tau, and p‐Tau181, did not significantly add information to the model.

**Conclusions:**

CSF biomarkers measured with the LUMIPULSE system showed good agreement with amyloid PET imaging. The ratio of Aβ42 with the other analyzed biomarkers showed a higher correlation with amyloid PET than Aβ42 alone, suggesting that the combinations of biomarkers could be useful in the diagnostic assessment in clinical research and potentially in routine clinical practice.

## Introduction

Dementia is not one specific disease but rather a group of symptoms, including memory loss, disorientation, confrontational behavior, language problems, a variety of behavioral, and psychological symptoms, which are accompanied by signs of deterioration of different brain functions. Alzheimer's disease (AD) is the most common cause of dementia in elderly people, and amyloid plaques and neurofibrillary tangles (NFTs) arising in the brain are the hallmarks of AD. Individuals with AD typically have no apparent cognitive decline and remain asymptomatic for one to two decades during the preclinical phase in which there is elevated brain Aβ burden.^1^, ^2^ As the disease progresses, individuals enter the symptomatic phase when their cognitive ability declines. In the mild cognitive impairment (MCI) stage, the impairment in different brain functions does not affect usual daily activities, whereas, in dementia stage, it can significantly interfere with a person's daily activities.

AD biomarkers have been used in clinical practice and clinical trials. Regarding clinical utility, increasing evidence points out that there are some discrepancies between the diagnosis made only with clinical symptoms and the result based on AD biomarkers; hence biomarkers can be used to enhance confidence that individuals are accurately diagnosed with AD.^3^, ^4^ Cerebrospinal fluid (CSF) biomarkers are especially useful when the etiology of cognitive impairment is uncertain, and AD is a possible cause. Clinical trials for AD drugs now routinely test CSF or positron emission tomography (PET) imaging biomarkers to confirm brain β‐amyloid (Aβ) pathology in potential participants, whereas, in the past AD drug trials, biomarkers were not used to select participants, which meant that inappropriate participants might have been enrolled.^5^, ^6^

In 2018, a taskforce organized by the National Institutes of Aging and the Alzheimer's Association (NIA‐AA) proposed a biomarker‐based diagnosis of AD based on the ATN classification system, which utilizes markers that reflect the main pathological hallmarks of AD: deposition of Aβ (Α), pathological Tau (T), and neurodegeneration (N).^7^ Amyloid PET and CSF biomarkers, especially β‐amyloid 1‐42 (Aβ42), can be used as “A” in that framework.

Molecular imaging biomarkers have become well established.^8^ Radiolabeled probes that bind to amyloid plaques (e.g., ^11^C‐Pittsburgh Compound B [^11^C‐PIB], ^18^F‐florbetapir, ^18^F‐florbetaben, ^18^F‐flutemetamol, and ^18^F‐NAV4694) can visualize amyloid plaques with PET. Although these PET imaging techniques provide information in terms of the amount and spatial distribution of brain Aβ pathology, several factors including high cost, limited availability, use of radiation, and imaging of only a single type of pathology per scan, limit their widespread use.^9^, ^10^

On the other hand, CSF biomarkers, especially Aβ 1‐40 (Aβ40), Aβ42, total tau (t‐Tau), and phosphorylated tau‐181 (p‐Tau181), can be used in the AD field to evaluate the level of different AD pathology‐related biological analytes and are more accessible and less expensive than amyloid PET. However, these markers have not been used routinely and widely. One of the reasons is the considerable hesitation in requesting lumbar puncture (LP) for AD diagnosis in several countries such as the United States and Japan.^11^ Such hesitation may be attributable to the perceived invasiveness of the LP procedure and its potential adverse events, such as postdural‐puncture headache.^11^ These adverse events can be alleviated by using atraumatic (pencil‐point) LP needles which, compared to conventional LP needles, reportedly reduce the risk of postdural‐puncture headache.^12^ The measurement accuracy might be another issue hampering the use of CSF markers. It has been pointed out that coefficients of variation (CVs) were too high in ELISA or xMAP assays.^13^ There are three factors that can affect the accuracy in measurements: (1) pre‐analytical (i.e., differences in the collection, handling, and storage of CSF),^14^, ^15^ (2) analytical (i.e., differences in technician skill or how the assays are used in the lab, and lot‐to‐lot variability of reference materials, kits and/or kit components),^16^, ^17^ and (3) biological/patient‐related (i.e., confounding factors linked to patients, such as patient age). Recently, a workgroup led by the Alzheimer's Association with experts from both academia and industry, proposed a simplified, and standardized pre‐analytical protocol for CSF collection and handling before analysis for routine clinical use to ensure high diagnostic performance and to minimize patient misclassification rates.^15^ Moreover, there have been several efforts to reduce the analytical variability. Automated assay platforms are developed to overcome some of these measurement drawbacks. FUJIREBIO Inc. has developed a chemiluminescent enzyme immunoassay (CLEIA) that utilizes the fully automated LUMIPULSE analyzers.^18^, ^19^, ^20^ This assay platform exhibits high degrees of precision, accuracy, reliability, and reproducibility in large part due to its automation.

To date, a number of studies have evaluated the relationship between CSF AD biomarkers and amyloid PET imaging, and found a strong correlation between the signal of amyloid PET ligands and the levels of CSF Aβ42, especially the ratio of Aβ42 with other AD biomarkers (e.g., t‐Tau/Aβ42, p‐Tau181/Aβ42, or Aβ42/Aβ40),^10^, ^18^, ^19^, ^21^, ^22^, ^23^ although t‐Tau/Aβ42 or p‐Tau181/Aβ42 ratios might lead to false positives because t‐Tau or p‐Tau181 could be upregulated by non‐AD tauopathies.^24^, ^25^ Furthermore, CSF Aβ42 reportedly detects brain Aβ deposition status earlier than amyloid PET.^21^, ^26^

In this study, we tested LUMIPULSE assay kits using CSF samples obtained from individuals who had undergone amyloid PET imaging as part of the screening process to establish eligibility for a clinical research program. We measured CSF Aβ40, Aβ42, t‐Tau, and p‐Tau181 sequentially with their respective LUMIPULSE assay reagents, and then examined the relationship between the status of brain Aβ pathology confirmed by amyloid PET visual read and CSF Aβ40, Aβ42, t‐Tau, and p‐Tau181, and the combinations of these markers.

## Materials and Methods

### Study population

Our cohort consisted of 199 subjects who had been enrolled in a clinical study (E2609‐G000‐301 or E2609‐G000‐302) conducted by Eisai and whose amyloid PET results and CSF samples were available.^27^ The subjects selected for this study had been clinically diagnosed as AD according to the core clinical criteria of diagnosis of MCI or dementia as defined in the 2011 NIA‐AA guidelines. During the screening phase of the clinical studies, all the subjects underwent amyloid PET scans to confirm elevated levels of brain Aβ deposition, which means that the sample set included both amyloid PET positive and negative subjects. Approvals for the clinical studies (E2609‐G000‐301 or E2609‐G000‐302) were obtained from the local ethics committees or institutional review committees, and all participants gave written consent. The ethics committee of Eisai Co. approved all the procedures of this biomarker study (REP‐2019‐0562‐013‐E).

### Amyloid PET imaging

Brain Aβ deposition was visualized using ^18^F‐florbetapir, ^18^F‐florbetaben, or ^18^F‐flutemetamol. Amyloid PET was conducted in accordance with predefined procedures as mentioned previously.^27^ Briefly, trained and formally qualified readers interpreted the images by visual read. The visual reads required agreement between two independent readers applying the standard procures of the tracers' manufacturers.

### CSF biomarkers

CSF samples from previous clinical studies (E2609‐G000‐301 or E2609‐G000‐302) were used in this study.^27^ Aβ40, Aβ42, t‐Tau, and p‐Tau181 were sequentially measured from the same sample aliquot using LUMIPULSE assay kits (FUJIREBIO Inc., Tokyo, Japan).^18^, ^19^, ^20^ The LUMIPULSE systems, for example, LUMIPULSE G1200, are chemiluminescence immunoassays using quantitative sandwich principle with a total assay duration of 30 min. Samples were tested according to the instructions for use. The measurements were carried out on the LUMIPULSE G1200 automated immunoassay analyzer with a single calibration run. Each assay required the following sample volume; 40 μL for Aβ40, 50 μL for Aβ42, 75 μL for t‐Tau and 40 μL for p‐Tau181. For each analyte, a single lot for assays was used to measure all the samples.

### Statistical analysis

Subject baseline characteristics were summarized using descriptive statistics. Categorical variables were presented as frequencies and percentages. Continuous variables were summarized into median, first quartile (Q1), and third quartile (Q3). Group comparisons for continuous variables were performed using the Wilcoxon rank‐sum test. Fisher's exact test was used for categorical data. All tests were done under the nominal significance level α = 0.05. Receiver operating characteristic (ROC) analyses were performed to determine the cutoffs and the performance of individual biomarkers and their combinations for the discrimination between amyloid PET positive and amyloid PET negative subjects. Cutoffs were derived using the Youden Index. AUC differences were assessed with DeLong's test.^28^ The univariate linear relationship between biomarkers was analyzed with Spearman's rank correlation. A univariate logistic regression model was used to calculate the odds ratio (OR) with a 95% confidence interval (CI) for the prediction of amyloid PET status. A multivariate logistic regression model was also used to adjust for other covariates: MMSE, sex, Aβ40, Aβ42, t‐Tau, and p‐Tau181. All statistical analyses were performed with R version 4.05 (R Foundation for Statistical Computing, Vienna, Austria). A two‐sided *p* < 0.05 was considered statistically significant.

## Results

### Participant characteristics

Table 1 summarizes the subject demographic characteristics and biomarker results. Almost all subjects (*n* = 198, 99.5%) had a clinical dementia rating (CDR) global score of 0.5, and one subject had a CDR‐global score of 1. The median intervals between CSF collection and amyloid PET imaging in amyloid PET negative and positive group were 10.5 days and 8.0 days, respectively, which was not significantly different. Eighty‐three subjects (41.7%) were amyloid PET‐positive. There were no differences in age, sex, clinical stage, or race between amyloid PET‐negative and amyloid PET‐positive subjects. Likewise, the average interval between CSF collection and amyloid PET imaging of the two groups was not significantly different.

**Table 1:** Clinical and demographic characteristics of all participants by amyloid PET status.

Characteristic	Amyloid negative	Amyloid positive	*p* value
*N* (%)	116 (58.3)	83 (41.7)	‐
Sex (%)*			1
F	52 (44.8)	37 (44.6)	‐
M	64 (55.2)	46 (55.4)	‐
Age Category (%)*			0.959
50 to <60	4 (3.4)	3 (3.6)	‐
60 to <70	33 (28.4)	25 (30.1)	‐
70 to <80	63 (54.3)	42 (50.6)	‐
80 to 85	16 (13.8)	13 (15.7)	‐
Amyloid PET Ligand (%)*			0.033
Florbetaben	87 (75.0)	66 (79.5)	‐
Florbetapir	5 (4.3)	9 (10.8)	‐
Flutemetamol	24 (20.7)	8 (9.6)	‐
Clinical Stage (%)*			0.053
MCI due to AD	103 (88.8)	81 (97.6)	‐
Mild AD	10 (8.6)	2 (2.4)	‐
ND	3 (2.6)	0 (0.0)	‐
Race (%)*			0.095
Caucasian	88 (75.9)	71 (85.5)	‐
Asian	24 (20.7)	8 (9.6)	‐
Others	4 (3.4)	4 (4.8)	‐
CDR‐global score (%)*			1
0.5	115 (99.1)	83 (100.0)	‐
1	1 (0.9)	0 (0.0)	‐
MMSE^†^	26 [25, 27]	27 [26, 28]	0.046
The interval between CSF collection and amyloid PET imaging^†^	10.5 [5.0, 17.0]	8.0 [6, 14.0]	0.752
Ab40, pg/ml^†^	13237 [11159, 15992]	13901 [11981, 16399]	0.146
Ab42, pg/ml^†^	1117 [839, 1404]	672 [540, 856]	<0.001
t‐Tau, pg/ml^†^	268 [224, 371]	535 [368, 779]	<0.001
p‐Tau181, pg/ml^†^	38.2 [30.4, 50.3]	77.3 [47.7, 117.9]	<0.001
Ab42/Ab40^†^	0.091 [0.075, 0.097]	0.048 [0.040, 0.054]	<0.001
t‐Tau/Ab42^†^	0.233 [0.190, 0.324]	0.810 [0.569, 1.214]	<0.001
p‐Tau181/Ab42^†^	0.030 [0.027, 0.042]	0.127 [0.078, 0.191]	<0.001

* Percent, *p* values by Fisher's exact test.

^†^ Median [Q1, Q3], *p* values by Mann–Whitney *U* test.

### Aβ40, Aβ42, t‐Tau, and p‐Tau181 concentrations on the LUMIPULSE G1200

**Figure 1:**
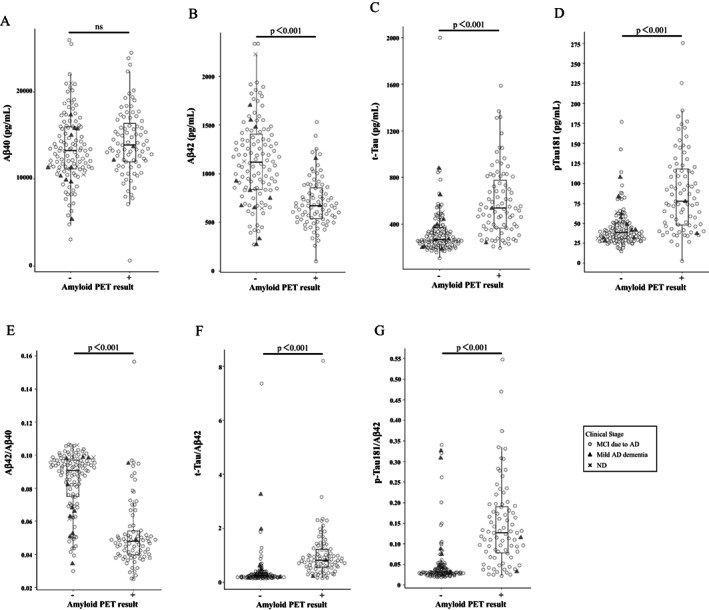
Distribution of biomarkers and biomarker ratios by amyloid positron emission tomography (PET)‐positive and ‐negative groups. Beeswarm boxplots of LUMIPULSE assays Aβ40 (A), Aβ42 (B), t‐Tau (C), p‐Tau181 (D), Aβ42/Aβ40 (E), t‐Tau/Aβ42 (F), and p‐Tau181/Aβ42 (G) by amyloid PET status. Box plots display the median values with the interquartile range (lower and upper hinge) and ± 1.5‐fold the interquartile range from the first and third quartile (lower and upper whiskers). Data were analyzed using Wilcoxon signed‐rank tests. ns: not significant.

The four biomarkers were measured in all 199 samples, but total‐Tau and p‐Tau181 concentrations of one sample could not be determined due to insufficient sample volume. The levels of the biomarkers in the analyzed samples ranged from 632 to 25,978 pg/ mL for Aβ40, 99 to 2,335 pg/ml for Aβ42, 107 to 2,000 pg/ml for t‐Tau, and 2.6 to 275.6 pg/ml for p‐Tau181. There were 2 and 1 samples that showed out‐of‐range values for Aβ42 and t‐Tau, respectively (Table 1; Fig. 1). Compared to amyloid PET‐negative subjects, amyloid PET‐positive subjects showed statistically significantly lower Aβ42 concentrations and higher t‐Tau and p‐Tau181 concentrations, but no statistically significant difference was observed for Aβ40 concentrations (Table 1, Fig. 1). When race‐based variations of these biomarkers were investigated, no significant differences were found (Table S1; Fig. S1). The Aβ42/Aβ40, t‐Tau/Aβ42, and p‐Tau181/Aβ42 ratios showed clearer differences between amyloid PET positive and negative subjects than single biomarkers. The *p*‐values of the statistically significant differences are presented as <0.001 (Fig. 1).

### CSF biomarker cutoffs for confirmation of brain Aβ pathology status

**Figure 2:**
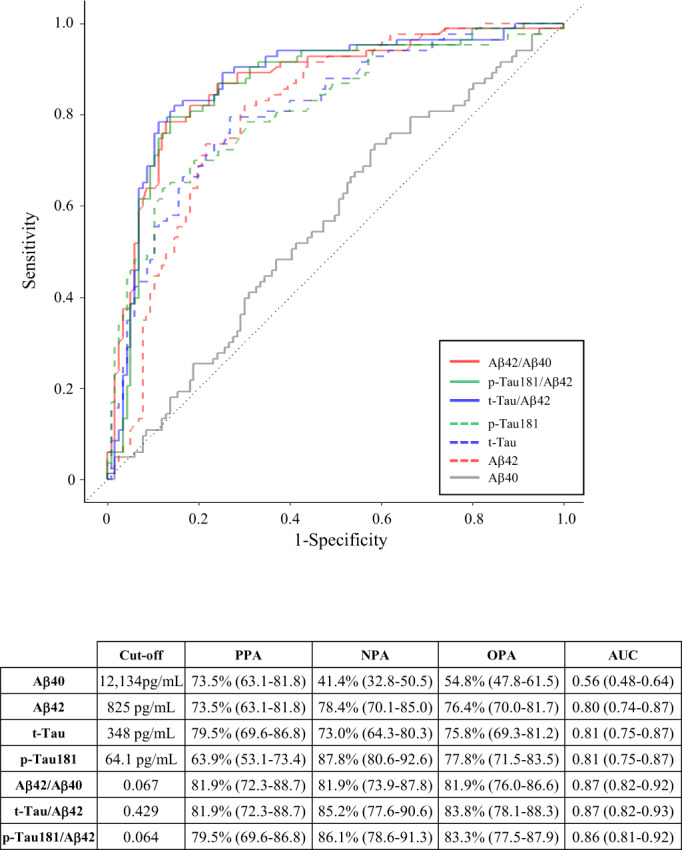
Receiver operator characteristic (ROC) curves for CSF biomarkers compared to amyloid PET results. For ROC analysis, individuals were dichotomized into amyloid PET‐negative and amyloid PET‐positive groups. For each CSF biomarker measure, the table indicates the cutoff values and associated positive percent agreement (PPA), negative percent agreement (NPA), overall percent agreement (OPA), and area under the ROC curve (AUC) for the measure compared to amyloid PET status. 95% confidence intervals are included in parentheses.

The ROC curves, the cutoff, the associated positive percent agreement (PPA), negative percent agreement (NPA), overall percent agreement (OPA) with amyloid PET status and area under the curve (AUC) of all biomarkers are shown in Figure 2. Aβ42, t‐Tau, and p‐Tau181 with AUC values of 0.80 (95% CI 0.74–0.87), 0.81 (95% CI 0.75–0.87), and 0.81 (95% CI 0.75–0.87), respectively, showed fair accuracy. There were no significant differences in accuracy between these biomarkers in this sample set. The p‐values for AUC comparison between Aβ42 and t‐Tau, Aβ42 and p‐Tau181, and, t‐Tau and p‐Tau181 were 0.96, 0.98, and 0.97, respectively. The combination of Aβ42 with other biomarkers significantly increased the accuracy. The AUC of Aβ42/Aβ40 was 0.87 (95% CI 0.82–0.92), which was higher than Aβ42 (*p* < 0.05) or Aβ40 alone (*p* < 0.001). t‐Tau/Aβ42 had an AUC of 0.87 (95% CI 0.82–0.93), higher than t‐Tau or Aβ42 alone (both *p* < 0.01), and p‐Tau181/Aβ42 had an AUC of 0.86 (95% CI 0.81–0.92), higher than pTau181 or Aβ42 alone (both *p* < 0.05). There were no significant differences in AUCs between Aβ42/Aβ40, t‐Tau/Aβ42, and p‐Tau181/Aβ42 ratios and adding a third biomarker to these models or a generalized linear model of these markers did not improve their accuracy (data not shown).

Since brain autopsy, but not amyloid PET imaging, is the gold standard for the confirmation of brain Aβ deposition, we referred to PPA and NPA rather than sensitivity and specificity, to evaluate CSF biomarker performances. As displayed in Figure 2, the cutoff of Aβ40, Aβ42, t‐Tau, and p‐Tau181 as determined using the Youden Index, were 12,134 pg/ml, 825 pg/ml, 348 pg/ml, and 64.1 pg/ml, respectively. For the Aβ42/Aβ40, t‐Tau/Aβ42, and p‐Tau181/Aβ42 combinations, the plots showed plateau stages indicating that a wide range of cutoffs yielded similar Youden indices. The best cutoffs were 0.067 for Aβ42/Aβ40, 0.429 for t‐Tau/Aβ42, and 0.064 for p‐Tau181/Aβ42. For Aβ40, Aβ42, t‐Tau, and p‐Tau181, the OPA values were 54.8%, 76.4%, 75.8%, and 77.8%, respectively. The ratio of Aβ42 with Aβ40, t‐Tau, or p‐Tau181 increased the OPA to 81.9%, 83.8%, and 83.3%, respectively.

### The logistic regression model to predict brain Aβ pathology status with CSF biomarkers or other factors

Univariate and multivariate logistic regression analyses were performed to find out which factors significantly contributed to the prediction of brain Aβ pathology status confirmed by amyloid PET. Table 2 contains ORs of the covariates associated with amyloid PET positivity and 95% CIs. The result of multivariate logistic regression analyses showed that amyloid PET status was associated with Aβ40, and Aβ42, with an adjusted OR of 1.0003 for Aβ40 (95% CI: 1.0001–1.0005, *p* < 0.01), and 0.9951 for Aβ42 (95% CI: 0.9930–0.9969, *p* < 0.001). However, other factors, such as MMSE, sex, t‐Tau, and p‐Tau181, did not significantly add information to the model after adjustments for all potential confounding factors. The variance inflation factor (VIF) was calculated to evaluate the possibility of multicollinearity. VIF values ranged from 1.07 to 3.44 (Table S2), suggesting that there was no strong multicollinearity.

**Table 2:** Odds ratios (OR) and 95% confidence intervals (95% CI) of amyloid PET positivity associated with covariates.

Variable	Univariate			Multivariate		
	OR	95% CI	*p* value	OR	95% CI	*p* value
MMSE	1.2070	1.0084–1.4526*	0.0425	1.2313	0.9557–1.6032	0.1124
Sex	1.0101	0.5731–1.7842	0.9720	0.8642	0.3855–1.9206	0.7200
Ab40	1.0000	1.0000–1.0001	0.2320	1.0003	1.0001–1.0005**	0.0020
Ab42	0.9964	0.9952–0.9975***	0.0000	0.9951	0.9930–0.9969***	<0.0001
t‐Tau	1.0047	1.0032–1.0065***	0.0000	1.0002	0.9981–1.0025	0.8730
p‐Tau181	1.0361	1.0250–1.0491***	0.0000	1.0072	1.0072–1.0283	0.4721

* *p* < 0.05.

** *p* < 0.01.

*** *p* < 0.001.

### Discordance between CSF biomarker combinations and amyloid PET status

**Figure 3:**
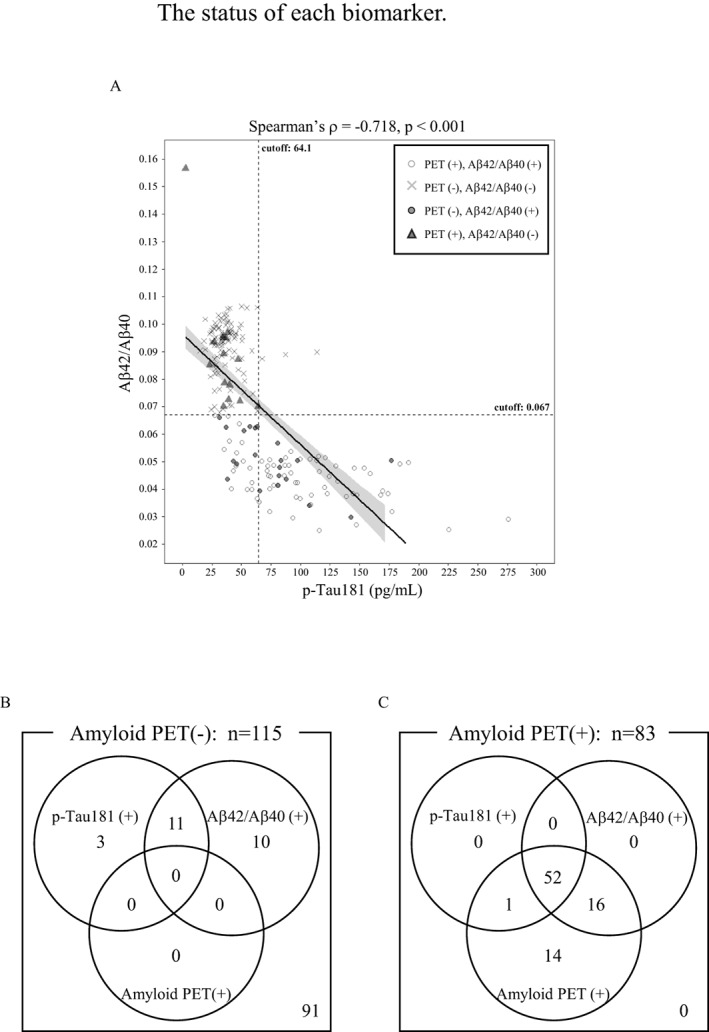
The status of each biomarker. (A) Correlation plots between Aβ42/Aβ40 and p‐Tau181 with superimposed the linear regression lines with 95% confidence intervals. Positive or negative according to CSF ratios or amyloid PET results was evaluated in scatterplot. The vertical and horizontal dashed lines represent cutoffs for p‐Tau181 and Aβ42/Aβ40, respectively. The statistical methods of Spearman's rank correlation coefficient (ρ) were used. p‐value is indicated. Solid line, linear regression lines; open circle, amyloid PET positive and CSF Aβ42/Aβ40 ratio positive; cross, amyloid PET negative and CSF Aβ42/Aβ40 ratio negative; filed circle, amyloid PET negative and CSF Aβ42/Aβ40 ratio positive; triangle, amyloid PET positive and CSF Aβ42/Aβ40 ratio negative. (B, C) Venn's diagrams show the performance of the biomarkers positivity in amyloid PET negative group (B) and positive group (C). Numbers indicate the intersection positivity of the biomarkers.

We used the Aβ42/Aβ40 ratio for further study and investigated the cases of discordance between CSF Aβ42/Aβ40 ratio and amyloid PET results. CSF Aβ42/Aβ40 ratio was negative in 15 (18%) of the 83amyloid PET‐positive individuals, and Aβ42/Aβ40 ratio was positive in 21 (18%) of the 116 amyloid PET‐negative individuals (Fig. 3). An Aβ42/Aβ40 ratio versus p‐Tau181 scatterplot was constructed to assess the biomarker‐based status of these discordant cases (Fig. 3A). These data suggest that Aβ42/Aβ40 ratio substantially correlated with p‐Tau181 (Spearman's ρ = −0.718, *p* < 0.001). Based on the cutoff points defined in Figure 2, Aβ42/Aβ40 ratio and p‐Tau181 were positive in 11 (52%) of the 21 discordant amyloid PET‐negative individuals (Fig. 3A,B). There were 3 cases which were p‐Tau181 positive but neither their Aβ42/Aβ40 ratio nor amyloid PET results were positive (Fig. 3B). Total‐Tau was also abnormal in these cases. In 14 (93%) of the 15 discordant amyloid PET‐positive individuals, both Aβ42/Aβ40 ratio and p‐Tau181 biomarkers were negative (Fig. 3A,C). The three CSF biomarker combinations (Aβ42/Aβ40, t‐Tau/Aβ42, and p‐Tau181/Aβ42) performed well in discriminating amyloid PET‐positive and PET‐negative individuals, and in the amyloid PET‐positive and Aβ42/Aβ40‐negative cases, t‐Tau/Aβ42 and p‐Tau181/Aβ42 patterns were similar patterns to that of Aβ42/Aβ40 (Fig. S2). On the other hand, among the 21 amyloid PET‐negative and Aβ42/Aβ40‐positive cases, only 6 individuals were negative for t‐Tau/Aβ42 and p‐Tau181/Aβ42 ratio (Fig. S2).

## Discussion

In our study, we determined the cutoffs for four CSF AD biomarkers (Aβ40, Aβ42, t‐Tau, and p‐Tau181) and their ratios measured on the fully automated LUMIPULSE G1200 platform to optimize their concordance with the brain Aβ pathology status determined by amyloid PET visual read. Consistent with previous reports,^18^, ^19^, ^20^, ^23^, ^27^, ^29^ we found that Aβ42/Aβ40, t‐Tau/Aβ42, and p‐Tau181/Aβ42 ratios had a better diagnostic agreement with amyloid PET results than single biomarkers. However, previous studies, including those performed on samples from the same clinical trial, reported cutoff values different from ours.^27^ Cutoff differences might be explained by differences in sample size, assay setting, or even the sample handling process after thawing. Further studies are required to address these points.

The multivariate logistic regression analyses indicated that amyloid PET status was significantly associated with CSF Aβ40 and Aβ42, whereas other factors, such as MMSE, sex, t‐Tau, and p‐Tau181, did not add significant information. Since t‐Tau or p‐Tau181 concentrations can be affected by non‐AD tauopathies,^24^, ^25^ the result of multivariate logistic regression analyses is plausible. These data suggest that CSF Aβ40 and Aβ42 combination is the preferred CSF AD biomarker for the prediction of brain Aβ pathology, though Aβ42/Aβ40 ratio might not always be the best model. Moreover, we showed the cutoffs of t‐Tau and p‐Tau181 as determined by amyloid PET status. In this study, there was a statistically significant correlation between CSF Aβ42/Aβ40 ratio and p‐Tau181 as shown in Figure 3A, suggesting that both CSF Aβ42/Aβ40 ratio and p‐Tau181 might be used for the prediction of brain Aβ pathology. Actually, it has been reported that plasma p‐Tau might serve as a predictive biomarker for brain amyloidosis.^30^, ^31^, ^32^ However, since t‐Tau and p‐Tau181 could reflect pathologies other than brain Aβ pathology, these cutoffs might not be appropriate for the confirmation of brain Aβ pathology status. A possible explanation for the disease‐based cutoff variation is the disease‐specific variations of tau phosphorylation sites.^33^ Indeed, it has been reported that other p‐tau isoforms, for example, p‐Tau217, might better reflect AD pathology.^34^ Furthermore, the specimens used in this study were from suspected MCI due to AD and mild AD dementia, but in routine clinical practice, the patients who are in non‐AD dementia and even the preclinical AD stages, could be included, suggesting the cutoffs could vary. Therefore, further investigations should address these points with appropriate groups fit for that purpose.

The agreement between amyloid PET imaging and CSF biomarkers has previously been studied.^18^, ^19^, ^22^, ^23^ Our results are consistent with previous studies which showed that ratios with Aβ42 had a higher overall agreement with amyloid PET status than single analytes. This suggests that the use of the ratios with Aβ42 could cancel the individual differences in Aβ pathophysiological process that would otherwise have led to false positive or false negative results. However, CSF biomarker‐specific cutoff points slightly differ between studies, and several methodological differences can explain these discrepancies. First, pre‐analytical conditions, such as the type of collection and storage tubes, differed between studies, and these factors are known to have a great impact on the absolute values of CSF biomarkers, especially for Aβ42.^14^, ^15^ Second, some analytical parameters, for example, specificity of the antibodies, time of incubation for each immunoassay and platform used in these studies resulted in diverse CSF biomarker measurements. The LUMIPULSE system had been recalibrated with certified reference material, but other assays might not have reflected the calibration in their measurement values yet. Third, differences in the properties of radiolabeled probes (^11^C‐PIB, ^18^F‐florbetapir, ^18^F‐florbetaben, or ^18^F‐flutemetamol) could generate different cutoffs because of their different uptake characteristics.^35^, ^36^ Moreover, we used PET visual read, but other studies used PET standard uptake value ratio (SUVR), which might lead to different cutoffs. To finish, the composition of the subject populations was not the same across studies. In our study, we included subjects with suspected MCI due to AD and Mild AD dementia from the clinical trial of disease‐modifying therapy for AD, which might more realistically reflect the application of biomarkers in daily clinical practice.

Various explanations have been given for the cases of discrepancy between amyloid PET imaging and CSF Aβ42/Aβ40 ratio test.^21^, ^26^, ^37^ The Aβ molecules detectable by amyloid PET imaging are insoluble aggregates (senile plaques), whereas the CSF Aβ42/Aβ40 ratio mainly confirms brain Aβ pathology by measuring soluble Aβ species, mainly Aβ monomers. This might cause certain discrepancies. In addition, the specimens used in this study were from suspected MCI due to AD and Mild AD dementia subjects, and some of them might be around the borderline of brain Aβ pathology. One of the explanations for the discordant cases between CSF Aβ42/Aβ40 ratio test and amyloid PET might be that the CSF Aβ42/Aβ40 ratio could detect an earlier change in Aβ pathology. Indeed, it has been reported that the discordant patients who had results of CSF Aβ42/Aβ40 ratio test positive and amyloid PET negative converted to amyloid PET positive within a few years.^26^ In this study, 11 of 21 patients with negative amyloid PET and positive Aβ42/Aβ40 ratio were in the abnormal range of CSF p‐Tau181 (Fig. 3). Moreover, in the amyloid PET negative and Aβ42/Aβ40 ratio positive cases, 6 out of 21 cases were positive in Aβ42/Aβ40 ratio but negative for both t‐Tau/Aβ42 and p‐Tau181/Aβ42 (Fig. S2). AD is an irreversible progressive disease, and early diagnosis and treatment are important because the therapeutic interventions being actively developed directly target AD pathology and are expected to be more effective in earlier stage. Therefore, the AD‐related markers in CSF would be useful as a diagnostic tool to provide accurate diagnosis and therapeutic opportunities to patients.

On the other hand, 15 discordant cases with amyloid PET‐positive and CSF Aβ42/Aβ40 ratio negative were identified. Among them, only 1 case was positive for p‐Tau181 in CSF (Fig. 3C), and its Aβ42/Aβ40 ratio was 0.070, which was around the borderline of the cutoff. The remaining 14 cases were p‐Tau181 negative, and one of them showed unexpectedly low values for Aβ40 (632 pg/ml), Aβ42 (99 pg/ml), and p‐Tau181 (2.6 pg/ml). It might be due to the presence of interfering substances in the sample, some contaminations such as proteases or handling issue, but, unfortunately, we were unable to perform further examinations due to the limited sample volume. Both the CSF Aβ42/Aβ40 ratio and p‐Tau181 of the other 13 cases, were negative, suggesting that CSF Aβ42/Aβ40 ratio might reflect the brain Aβ pathology more accurately. Actually, it has been reported that the causes of these discordances could be non‐specific uptake of the amyloid PET probe into the white matter, errors in the imaging process, over‐reading errors, or other factors.^26^ Longitudinal analyses of amyloid PET‐positive and CSF Aβ negative cases showed that the accumulation of Aβ in the brain did not increase and their clinical symptoms did not progress.^26^ To continue, several studies pointed out that, because it is necessary to consider factors such as the detection of Aβ aggregates that accumulate in blood vessel walls and partial volume effects in addition to senile plaques in the brain parenchyma, amyloid PET might not be a perfect method for the detection of Aβ pathology in AD.^38^, ^39^, ^40^ In this study, unfortunately, we were unable to obtain the follow‐up samples.

The International Working Group (IWG) recommended that the diagnosis of AD should be based on both clinical and biomarker evidence,^41^ because a purely biological definition of AD, positive Aβ, and tau markers, do not always predict that dementia will occur in a patient's lifetime. Moreover, AD biomarkers are commonly found in other neurodegenerative diseases. Therefore, IWG suggested that a comprehensive diagnosis combining clinical symptoms and other factors including biomarkers would be important.^41^

Recently, it has been reported that racial differences are present in some biomarkers of AD.^42^ Morris *et al*. showed that cognitively impaired African Americans with the *APOEε4* genotype, a risk factor for AD, had significantly lower levels of t‐Tau and p‐Tau181 than did Caucasians with similar conditions. On the other hand, there was no significant difference in the level of Aβ42.^42^ In this study, however, no racial differences in the four biomarkers were observed, but this might be due to the differences in sample sets, sample sizes, assay platforms, or these combinations. Only six African Americans (three amyloid PET positive and three amyloid negative subjects) were included in the sample set of this study, which was mainly composed of Caucasian and some dozens of Japanese. Further studies are required to confirm the racial difference issues.

Blood‐based AD biomarkers with new high‐sensitivity assays have emerged.^43^ The concentrations of Aβ and p‐Tau181 proteins in plasma correlate with their concentrations in CSF and with amyloid PET status.^31^, ^44^, ^45^ However, one issue with the blood‐based biomarkers might be the variations in assays outcomes reported by a cross‐sectional study examining the performance of several plasma assays for quantification of Aβ42/Aβ40.^45^ A major limitation of plasma tests (e.g., Aβ42/Aβ40) is that the biomarker levels are only decreased by 10%–20% in individuals with cerebral Aβ pathology, compared to 40%–60% for CSF Aβ42/40.^44^, ^46^, ^47^ As a result, plasma biomarker levels could be affected by small measurement variations caused by preanalytical handling and analytical performance, leading to misclassification (i.e., false‐negative or false‐positive for Aβ pathology). Nevertheless, blood tests could be used more broadly in the near future because they are minimally invasive and cost‐effective. Blood tests could be used as screening tools, and then positive patients would proceed to a specialized memory clinic for precise examination with CSF test or amyloid PET imaging.^48^

In this study, we found that the Aβ42 ratios with Aβ40, t‐Tau, or p‐Tau181 in CSF showed a substantial agreement with amyloid PET results and that the combinations of Aβ42 with other markers had a better correlation with the amount of amyloid PET results than Aβ42 alone. The multivariate logistic regression analyses indicated that amyloid PET status was significantly associated with CSF Aβ40 and Aβ42, but t‐Tau or p‐Tau181 did not substantively improve the predictive ability of the model, suggesting that using the Aβ42/Aβ40 ratio could predict the brain Aβ pathology more accurately and lead to an accurate diagnosis of AD. Moreover, the Aβ42/Aβ40 ratio test might detect brain Aβ pathology earlier than amyloid PET. This CSF Aβ42/Aβ40 ratio test therefore would be useful in the diagnostic assessment in clinical practice.

## The Strengths and Limitations of the Study

The strengths of this study are the simultaneous measurement of AD biomarkers in CSF with an automated system and the use of samples obtained from a multicenter population, including Caucasian and Asian participants. The limitations are the lack of a control group (cognitive normal) or a real‐world population in clinical practice that includes etiologies other than AD, as well as the lack of quantification of Aβ deposition. Also, selecting patients who were eligible for screening in clinical trials might have introduced a bias in this biomarker analysis. Moreover, we were unable to obtain the *APOE* genotype information. Furthermore, because of the lack of longitudinal data, it was not possible to construct and evaluate a model for predicting disease progression. Further studies will be carried out to provide a more direct assessment of the ability of CSF biomarkers to predict disease progression in clinical studies.

## Author Contributions

H.N. and K.A. designed the study. S.I. and D.V. provided samples and clinical data. A.A. and W.M. measured CSF samples. H.N. performed data analysis and statistical analysis. H.N. and S.I. prepared the manuscript. All authors participated in discussions and interpretation of the data and results.

## Conflict of Interest

H.N., A.K., A.A., W.M., and K.A. are employees of FUJIREBIO Inc. S.I. is an employee of Eisai Co., Ltd., and Eisai Inc. D.V. are employees of Eisai Inc. M.V., G.J., and I. V. are employees of Fujirebio‐Europe N.V. This study was funded by FUJIREBIO Inc.

## Supporting information


**Figure S1** Distribution of biomarkers among the races. (A‐D) Beeswarm boxplots of LUMIPULSE biomarkers in amyloid PET positive group. Aβ40 (A), Aβ42 (B), t‐Tau (C), and p‐Tau181 (D). (E‐H) Beeswarm boxplots of LUMIPULSE biomarkers in amyloid PET negative group. Aβ40 (E), Aβ42 (F), t‐Tau (G), and p‐Tau181 (H). Box plots display the median values with the interquartile range (lower and upper hinge) and ± 1.5‐fold the interquartile range from the first and third quartile (lower and upper whiskers). Data were analyzed using Kruskal–Wallis rank sum test and there were no significant differences.

**Figure S2** The status of each biomarker. (A, B) Correlation plots between Aβ42/Aβ40 and t‐Tau/Aβ42 (A) or between Aβ42/Aβ40 and p‐Tau181/Aβ42 (B) with superimposed linear regression lines with 95% confidence intervals. Positive or negative according to CSF ratios or amyloid PET results was evaluated in a scatterplot. The vertical dashed lines represent cutoffs for t‐Tau/Aβ42 (A) or p‐Tau181/Aβ42 (B), respectively. The horizontal dashed lines represent cutoffs for Aβ42/Aβ40. The statistical methods of Spearman's rank correlation coefficient (ρ) were used. p‐value is indicated. Solid line, Linear regression lines; open circle, amyloid PET positive and CSF Aβ42/Aβ40 ratio positive; cross, amyloid PET negative, and CSF Aβ42/Aβ40 ratio negative; siled circle, amyloid PET negative, and CSF Aβ42/Aβ40 ratio positive; triangle, amyloid PET positive, and CSF Aβ42/Aβ40 ratio negative. (C) Venn's diagrams show the performance of the biomarkers positivity in amyloid PET negative and positive groups. Numbers indicate the intersection positivity of the biomarkers.

**Table S1** The biomarker status differentiated by race. Median [Q1, Q3] shown in each group. *p* values by Kruskal–Wallis rank sum test.

**Table S2** The variance inflation factor (VIF) of each variable.
