# Supplementary figures and images for: Clinical utility of cerebrospinal fluid biomarkers measured by LUMIPULSE® system
Source: Ann Clin Transl Neurol. 2022 Nov 2;9(12):1898–909. doi: 10.1002/acn3.51681 (PMC9735374; doi:10.1002/acn3.51681)

Supplemental Figure 1: Distribution of biomarkers among the races.

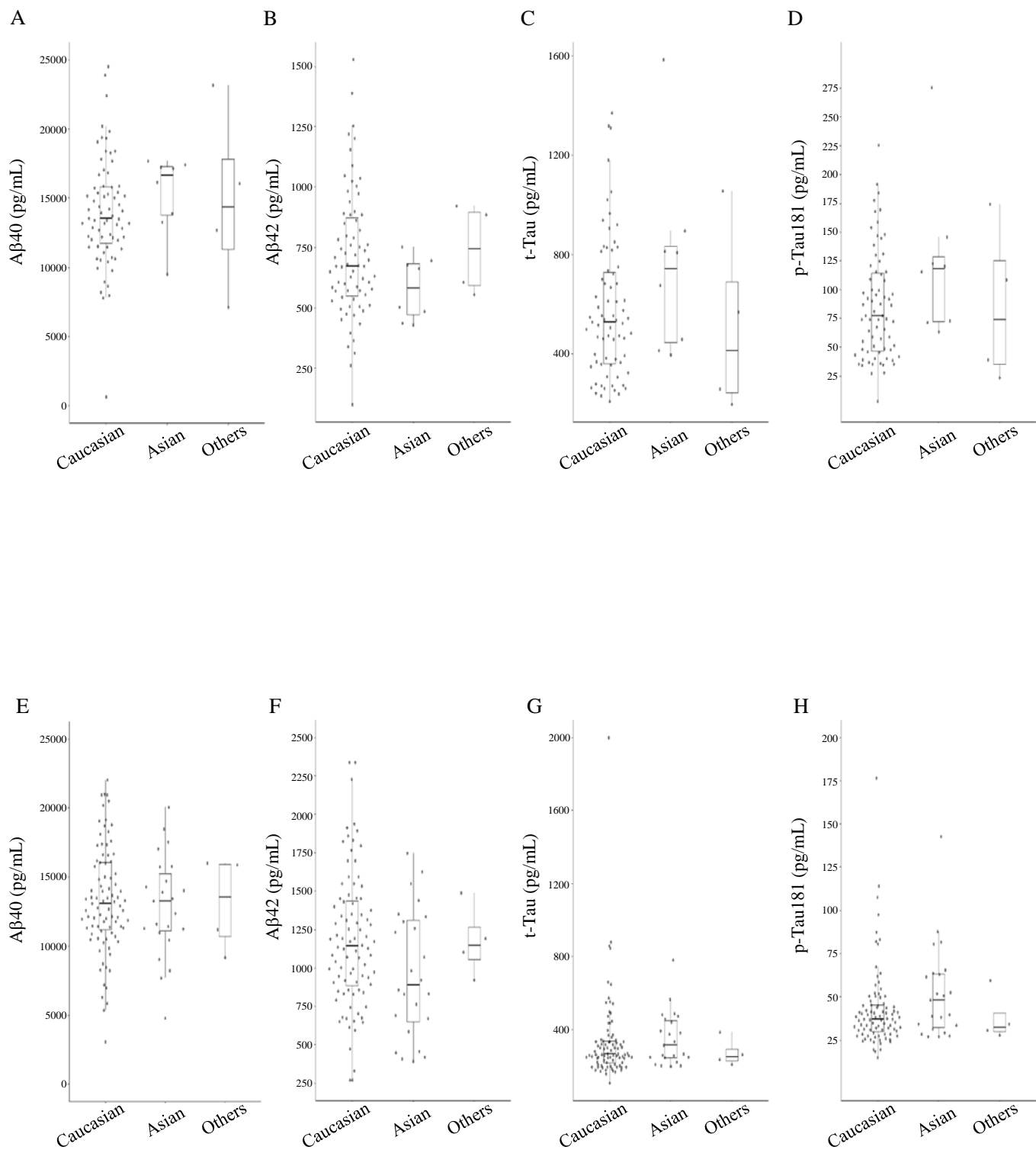

Supplement: Supplementary file 1 — Figure S1 Distribution of biomarkers among the races. (A‐D) Beeswarm boxplots of LUMIPULSE biomarkers in amyloid PET positive group. Aβ40 (A), Aβ42 (B), t‐Tau (C), and p‐Tau181 (D). (E‐H) Beeswarm boxplots of LUMIPULSE biomarkers in amyloid PET negative group. Aβ40 (E), Aβ42 (F), t‐Tau (G), and p‐Tau181 (H). Box plots display the median values with the interquartile range (lower and upper hinge) and ± 1.5‐fold the interquartile range from the first and third quartile (lower and upper whiskers). Data were analyzed using Kruskal–Wallis rank sum test and there were no significant differences. [file ACN3-9-1898-s003.pdf]

Supplemental Figure 2: : The status of each biomarker.

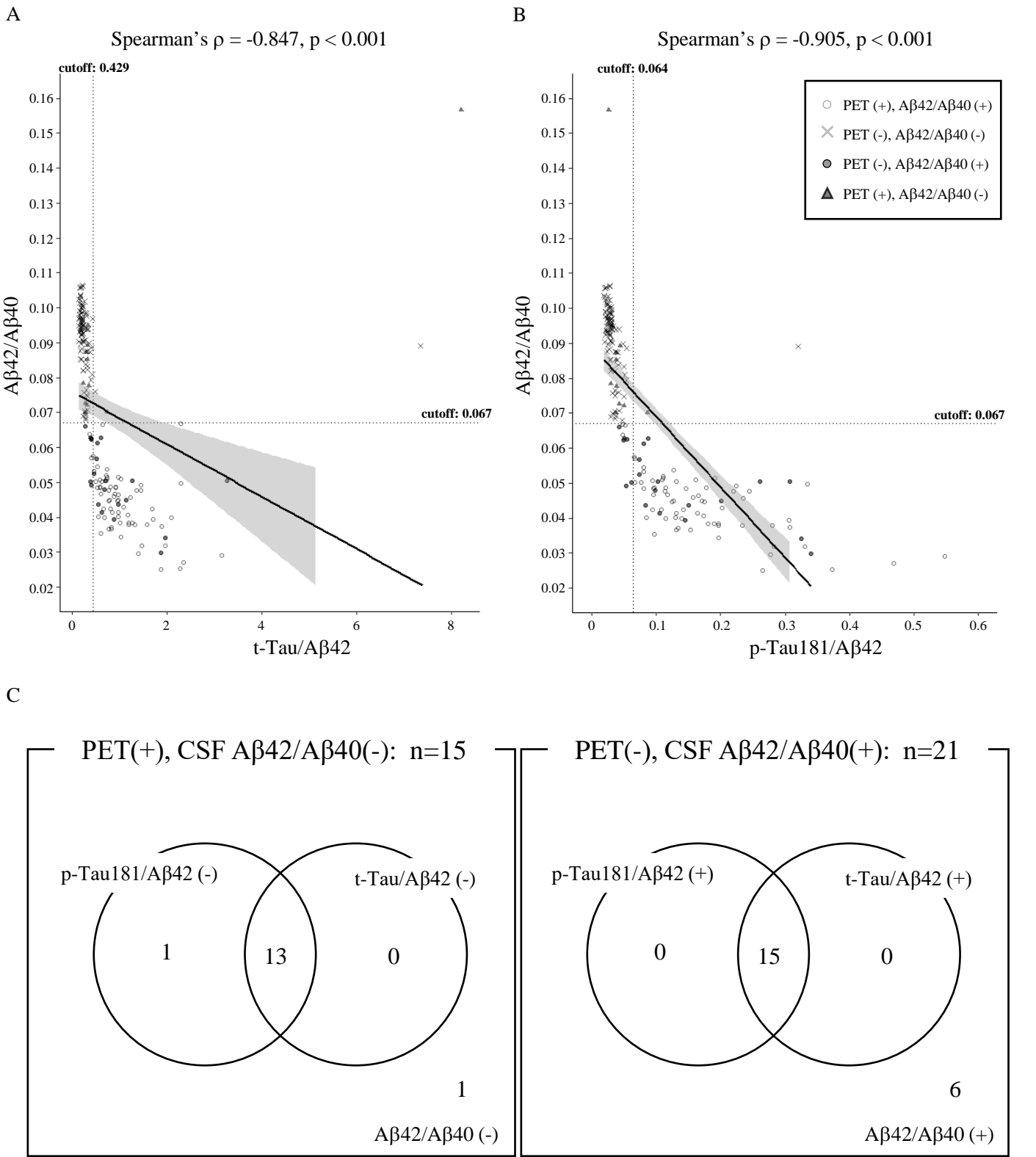

Supplement: Supplementary file 2 — Figure S2 The status of each biomarker. (A, B) Correlation plots between Aβ42/Aβ40 and t‐Tau/Aβ42 (A) or between Aβ42/Aβ40 and p‐Tau181/Aβ42 (B) with superimposed linear regression lines with 95% confidence intervals. Positive or negative according to CSF ratios or amyloid PET results was evaluated in a scatterplot. The vertical dashed lines represent cutoffs for t‐Tau/Aβ42 (A) or p‐Tau181/Aβ42 (B), respectively. The horizontal dashed lines represent cutoffs for Aβ42/Aβ40. The statistical methods of Spearman's rank correlation coefficient (ρ) were used. p‐value is indicated. Solid line, Linear regression lines; open circle, amyloid PET positive and CSF Aβ42/Aβ40 ratio positive; cross, amyloid PET negative, and CSF Aβ42/Aβ40 ratio negative; siled circle, amyloid PET negative, and CSF Aβ42/Aβ40 ratio positive; triangle, amyloid PET positive, and CSF Aβ42/Aβ40 ratio negative. (C) Venn's diagrams show the performance of the biomarkers positivity in amyloid PET negative and positive groups. Numbers indicate the intersection positivity of the biomarkers. [file ACN3-9-1898-s004.pdf]
