# Supplementary material for: Clinical utility of cerebrospinal fluid biomarkers measured by LUMIPULSE® system
Source: Ann Clin Transl Neurol. 2022 Nov 2;9(12):1898–909. doi: 10.1002/acn3.51681 (PMC9735374; doi:10.1002/acn3.51681)
Supplement: Supplementary file 3 — Table S1 The biomarker status differentiated by race. Median [Q1, Q3] shown in each group. p values by Kruskal–Wallis rank sum test. [file ACN3-9-1898-s002.pdf]

Supplemental Table 1: The biomarker status differentiated by race.

	Amyloid negative				Amyloid positive			
	Caucasian	Asian	Others	*p* value	Caucasian	Asian	Others	*p* value
Aβ40, pg/mL Median [Q1, Q3]	13140 [11260-16069]	13354 [11173-15746]	13532 [10679-15908]	0.9516	13508 [11739-15809]	16630 [13735-17259]	1435 [11279-17807]	0.442
Aβ42, pg/mL Median [Q1, Q3]	1143 [884-1436]	891 [650-1309]	1148 [1057-1267]	0.1358	672 [549-873]	582 [472-682]	745 [591-895]	0.2512
t-Tau, pg/mL Median [Q1, Q3]	267 [221-340]	314 [248-450]	250 [230-294]	0.4176	529 [362-729]	742 [447-834]	414 [242-691]	0.3473
p-Tau181, pg/mL Median [Q1, Q3]	37.2 [30.0-45.5]	48.1 [32.4-63.4]	32.5 [29.9-40.6]	0.1794	77.1 [46.7-114.5]	117.9 [72.4-128.2]	73.8 [35.1-124.9]	0.2455
