# Supplementary material for: Clinical utility of cerebrospinal fluid biomarkers measured by LUMIPULSE® system
Source: Ann Clin Transl Neurol. 2022 Nov 2;9(12):1898–909. doi: 10.1002/acn3.51681 (PMC9735374; doi:10.1002/acn3.51681)
Supplement: Supplementary file 4 — Table S2 The variance inflation factor (VIF) of each variable. [file ACN3-9-1898-s001.pdf]

**Supplemental Table 2:** The variance inflation factor (VIF) of each variable.

| Variable | VIF |
|---|---|
| MMSE | 1.14 |
| Sex | 1.07 |
| Aβ40 | 3.12 |
| Aβ42 | 2.66 |
| t-Tau | 2.21 |
| p-Tau181 | 3.44 |
